# An application in identifying high-risk populations in alternative tobacco product use utilizing logistic regression and CART: a heuristic comparison

**DOI:** 10.1186/s12889-015-1582-z

**Published:** 2015-04-09

**Authors:** Yang Lei, Nikki Nollen, Jasjit S Ahluwahlia, Qing Yu, Matthew S Mayo

**Affiliations:** Department of Biostatistics, The University of Kansas Medical Center, Kansas City, KS USA; Department of Preventive Medicine and Public Health, The University of Kansas Medical Center, Kansas City, KS USA; Department of Medicine and Center for Health Equity, The University of Minnesota Medical School, Minneapolis, MN USA

**Keywords:** Survey sampling, Stratified samples, Logistic regression, CART, Partial interaction

## Abstract

**Background:**

Other forms of tobacco use are increasing in prevalence, yet most tobacco control efforts are aimed at cigarettes. In light of this, it is important to identify individuals who are using both cigarettes and alternative tobacco products (ATPs). Most previous studies have used regression models. We conducted a traditional logistic regression model and a classification and regression tree (CART) model to illustrate and discuss the added advantages of using CART in the setting of identifying high-risk subgroups of ATP users among cigarettes smokers.

**Methods:**

The data were collected from an online cross-sectional survey administered by Survey Sampling International between July 5, 2012 and August 15, 2012. Eligible participants self-identified as current smokers, African American, White, or Latino (of any race), were English-speaking, and were at least 25 years old. The study sample included 2,376 participants and was divided into independent training and validation samples for a hold out validation. Logistic regression and CART models were used to examine the important predictors of cigarettes + ATP users.

**Results:**

The logistic regression model identified nine important factors: gender, age, race, nicotine dependence, buying cigarettes or borrowing, whether the price of cigarettes influences the brand purchased, whether the participants set limits on cigarettes per day, alcohol use scores, and discrimination frequencies. The C-index of the logistic regression model was 0.74, indicating good discriminatory capability. The model performed well in the validation cohort also with good discrimination (c-index = 0.73) and excellent calibration (R-square = 0.96 in the calibration regression). The parsimonious CART model identified gender, age, alcohol use score, race, and discrimination frequencies to be the most important factors. It also revealed interesting partial interactions. The c-index is 0.70 for the training sample and 0.69 for the validation sample. The misclassification rate was 0.342 for the training sample and 0.346 for the validation sample. The CART model was easier to interpret and discovered target populations that possess clinical significance.

**Conclusion:**

This study suggests that the non-parametric CART model is parsimonious, potentially easier to interpret, and provides additional information in identifying the subgroups at high risk of ATP use among cigarette smokers.

## Background

Recent years have witnessed increased tobacco control policies at both the state and national level [1-3]. Most of these efforts are aimed at cigarette smoking [1]. The net effects of these policies include decreased cigarettes consumption, as well as a shift in the type of tobacco products used [4,5]. The use of alternative forms of tobacco products (ATPs), such as large cigars, little cigars, cigarillos, pipes, hand-rolled cigarettes, smokeless tobacco, and hookahs, are increasing in prevalence [6,7]. About 8%-38% of U.S. daily smokers and as many as 44% of non-daily smokers (smoke on some but not all days) are ATP users [5-10], defined as anyone who uses cigarettes and alternative forms of tobacco. These tobacco products have been promoted as less addictive and less harmful than cigarettes [11,12]. Nevertheless, data suggest that use of these products could be associated with higher nicotine dependence and may contribute to increased risks for diseases caused by tobacco, such as cancer and heart disease [13].

It is of utmost importance to identify individuals who are at high risk of using both cigarettes and ATPs. Research subjects in previous studies have been predominately White [8,9,14,15] and most existing studies have used traditional regression approaches to identify important factors associated with ATP use. Although regression methods can test a priori specified interaction effects, it lacks the ability to capture unspecified, complex inter-relationships across factors. Classification and Regression Trees model (CART) can address these limitations by revealing unspecified inter-relationships through an easily interpretable tree diagram. Few studies have applied CART modeling to tobacco research [16,17]. In this paper we used data from a cross-sectional survey of smokers and conducted the most commonly used logistic regression method and relatively underused CART method, and described the strength and limitations of these two statistical approaches in identifying cigarette smokers at highest-risk for ATP use.

## Methods

### Study population

The data was collected through a cross-sectional survey administered through an online panel survey service, Survey Sampling International (SSI), between July 5, 2012 and August 15, 2012. Ethical approval was granted by the University of Minnesota Institutional Review Board. Participants were presented with a written informed consent page prior to completing the screener. Only participants who indicated their consent were directed to the study questions. Eligible participants self-identified as current smokers, African American, White, or Latino (of any race), were English-speaking, and were at least 25 years old. The study sample contained 2,376 participants balanced by the three racial/ethnic groups across smoking frequencies (daily and nondaily smoking): 794 African Americans, 786 Latinos, and 796 whites. Among them, 1,220 participants (51.35%) were cigarettes + ATP users who used both cigarettes and other tobacco products and 1,156 (48.65%) were cigarettes-only users. Variable domains in this study included: demographics, tobacco characteristics, cost concerns, harm reduction efforts, and psychosocial variables. There was minimal missing data, about 4.3% subjects were missing one variable (income), and therefore imputation was not necessary. Chi-square tests were used to test the unadjusted effects of categorical variables and T-tests were used to test continuous variables (Table 1).

**Table 1:** **Univariate differences between smokers who use cigarettes in combination with alternative tobacco product (cigarettes + ATP) compared to those who use cigarettes only**

	**Cigarettes + ATP**	**Cigarettes only**	**p value**
	**(n=1,220)**	**(n=1,156)**	
*Demographics*			
Male	662 (27.9%)	332 (14.0%)	<0.001
Age (±SD)	40.24 ± 11.64	45.85 ± 12.62	<0.001
Race			<0.001
African American	436 (18.4%)	358 (15.1%)	
Latino	455 (19.1%)	331 (13.9%)	
White	329 (13.8%)	467 (19.7%)	
Education, % college graduate or higher	474 (19.9%)	364 (15.3%)	<0.001
Income, % < $1800/month	480 (20.2%)	463 (19.5%)	0.725
*Tobacco Characteristics*			
Smoking status (%)			<0.001
Nondaily	673 (28.3%)	528 (22.2%)	
Daily light (1–10 cpd)	259 (10.9%)	319 (13.4%)	
Daily heavy (11+ cpd)	288 (12.1%)	309 (13.0%)	
Menthol smoker	737 (31.0%)	623 (26.2%)	0.001
Cigarettes per day, mean (±SD)	9.30 ± 8.70	10.14 ± 8.52	0.017
Time to first cigarette, % within 30 minutes of waking	720 (30.3%)	629 (26.5%)	0.024
24 hour quit attempts in last 12 months, mean (±SD)	5.50 ± 9.53	5.94 ± 11.79	0.451
*Cost*			
Price of cigs influenced them to smoke less, % yes	726 (30.6%)	644 (27.1%)	0.061
Price of cigs influenced where they buy cigs, % yes	840 (35.4%)	826 (34.8%)	0.166
Price of cigs influenced the brand they buy, % yes	590 (24.8%)	455 (19.1%)	<0.001
Buy versus borrow cigs, % buy all cigs they smoke	683 (28.7%)	824 (34.7%)	<0.001
*Harm Reduction*			
Trying to cut down on cigs smoke, % yes	862 (36.3%)	818 (34.4%)	0.955
Limit cpd to decrease health risk, % yes	596 (25.1%)	505 (21.3%)	0.012
Limit smoking in last year to decrease health risks, % always or often	360 (15.2%)	356 (15.0%)	0.494
*Psychosocial*			
Depression score, mean (±SD)^a^	2.14 ± 1.83	1.80 ± 1.84	<0.001
Alcohol score, mean (±SD)^b^	4.64 ± 3.10	3.30 ± 2.98	<0.001
Discrimination score, mean (±SD)^c^	8.28 ± 6.72	5.85 ± 5.66	<0.001

^a^Scores range from 0–6 with scores of 3 or higher indicating possible depressive symptoms.

^b^Scores range from 0–12 with scores of ≥4 for men and ≥3 for women indicating possible alcohol misuse.

^c^Scores range from 0–25 with higher scores indicating greater frequency of discrimination in daily life.

### Training and validation data sets

The large sample size allowed for the use of a hold-out validation to obtain independent training and validation data sets [18-23]. The data was partitioned by random sampling, stratifying by cigarettes + ATP use and race/ethnicity to ensure the balance we designed. Training sample contained 1,584 participants (two thirds of the sample) and was used to derive the model. The remaining data contained 792 participants (one third of the sample) and were used to evaluate the predictive ability of the final model. The training and validation samples were compared to ensure the differences between the two were negligible (Table 2).

**Table 2:** **Univariate Differences between training sample and validation sample**

	**Training**	**Validation**	**P value**
	**(n = 1584)**	**(n = 792)**	
*Demographics*			
Male	657 (27.7%)	337 (14.2%)	0.617
Age (±SD)	42.94 ± 12.39	43.03 ± 12.5	0.880
Race			0.997
African American	530 (22.3%)	264 (11.1%)	
Latino	524 (22.1%)	262 (11.0%)	
White	530 (22.3%)	266 (11.2%)	
Education, % college graduate or higher	550 (23.1%)	288 (12.1%)	0.430
Income, % < $1800/month	614 (25.8%)	329 (13.8%)	0.192
*Tobacco Characteristics*			
Smoking status (%)			0.263
Nondaily	799 (33.6%)	402 (16.9%)	
Daily light (1–10 cpd)	373 (15.7%)	205 (8.6%)	
Daily heavy (11+ cpd)	412 (17.3 )	185 (7.8%)	
Menthol smoker	899 (37.8%)	461 (19.4%)	0.500
Cigarettes per day, mean (±SD)	10.03 ± 9.03	9.06 ± 7.69	0.009
Time to first cigarette, % within 30 minutes of waking	900 (37.9%)	449 (18.9%)	0.953
24 hour quit attempts in last 12 months, mean (±SD)	5.54 ± 9.87	6.00 ± 11.93	0.454
*Cost*			
Price of cigs influenced them to smoke less, % yes	920 (38.7%)	450 (18.9%)	0.557
Price of cigs influenced where they buy cigs, % yes	1100 (46.3%)	566 (23.8%)	0.311
Price of cigs influenced the brand they buy, % yes	685 (28.8%)	360 (15.2%)	0.306
Buy versus borrow cigs, % buy all cigs they smoke	1004 (42.3%)	503 (21.2%)	0.952
*Harm Reduction*			
Trying to cut down on cigs smoke, % yes	1119 (47.1%)	561 (23.6%)	0.924
Limit cpd to decrease health risk, % yes	730 (30.7%)	371 (15.6%)	0.727
Limit smoking in last year to decrease health risks, % always or often	476 (20.0%)	240 (10.1%)	0.899
*Psychosocial*			
Depression score, mean (±SD)^a^	1.99 ± 1.86	1.96 ± 1.82	0.683
Alcohol score, mean (±SD)^b^	4.02 ± 3.16	3.93 ± 3.03	0.494
Discrimination score, mean (±SD)^c^	7.03 ± 6.30	7.23 ± 6.44	0.460

^a^Scores range from 0–6 with scores of 3 or higher indicating possible depressive symptoms.

^b^Scores range from 0–12 with scores of ≥4 for men and ≥3 for women indicating possible alcohol misuse.

^c^Scores range from 0–25 with higher scores indicating greater frequency of discrimination in daily life.

### Analysis

#### Logistic regression

Logistic regression is a traditional way to identify important factors for binary outcomes. The Akaike Information Criterion (AIC) is widely recommended as a model selection criterion [18]. To avoid the technical difficulty of comparing AICs from all possible variable combinations, we followed a model selection strategy recommended by Frank E. Harrell to trim the potential models [18] and then picked the minimal AIC model from the potential models as the final model. The selection process started with all potential factors. Predicted values from the logistic regression were then regressed on all covariates, with the model explaining 100% of the variance. Backward selection based on *R*^2^ was used to select a parsimonious set of variables. The contribution of each covariate in the multivariable model was ranked, and variables with the smallest contribution to the model were sequentially eliminated. This iterative process continued until further variable elimination led to a greater than 5% loss in model prediction, as compared with the initial model. The remaining covariates comprised the parsimonious model and explained over 95% of the variance of the full model. Finally, we compared AIC values of neighborhood models around the model we obtained in the last step and the minimum AIC model was selected as the final model. This selection strategy supports inclusion of only variables that provide incremental prognostic value, avoids over-fitting, and maximizes the potential usefulness of the model. Besides this model selection strategy, we examined backward selections based on p-value with 0.15 as the threshold to enter and 0.05 as the threshold to stay in the model. Both approaches identified the same model.

Predicted values using the model estimates from the training cohort were generated for the validation cohort and the c-index was then calculated based on the proportion of concordance. The predicted values were ranked and cut into deciles. The calibration plot was graphed comparing the average predicted probabilities with the observed average probabilities. A calibration regression on observed mean probabilities was performed using predicted mean probabilities to check the strength of correlation between the predicted and the observed average probabilities across deciles.

#### Classification and Regression Tree (CART) model

Although the logistic regression model provides knowledge of important profile characteristics, it lacks the ability to identify unknown, and therefore, unspecified interaction effects. The interpretation of parameter estimates is based on the fact of controlling for all other covariates. To address these problems, we built Classification and Regression Tree models (CART) in SAS Enterprise Miner version 12.3 [24,25]. CART is a nonparametric method that identifies mutually exclusive and exhaustive subgroups. Members within each subgroup share the same characteristics that influence the probability of belonging to the interested response group [26]. CART produces a model structure that resembles an upside-down tree. The tree starts with the parent node, and the parent node contains the entire population. The CART algorithm examines all possible independent variables according to a predetermined splitting rule and divides the parent node into two child nodes; the child nodes can be further divided into more child nodes. There are many splitting rules, and they all begin with defining the impurity of a node [26]. The impurity function measures the extent of difference/similarity for a node containing data points from possible different classes. A node that has no impurity would have no variability (e.g. all cigarettes-only smokers, or all cigarettes + ATP smokers). The highest impurity is achieved when p(k|t) = 0.5, where p(k|t) is defined as the conditional probability of belonging to class k given in node t. Although the impurity functions may vary, all splitting rules select the split that has the largest difference between the impurity of the parent node and a weighted average of the impurity of the two child nodes. The Gini splitting rule was recommended most for binary outcomes [25]. This rule maximizes the following improvement of impurity function:

$$ \overset{ \arg \kern0.5em  \max }{x_j\le {x}_j^R,j=1,\dots, M}\left[-{\displaystyle \sum_{k=1}^K{p}^2\left(\left.k\right|{t}_p\right)+{P}_l}{\displaystyle \sum_{k=1}^K{p}^2}\left(k\left|{t}_l\right.\right)+{P}_r{\displaystyle \sum_{k=1}^K{p}_2}\left(k\left|{t}_r\right.\right)\right] $$

p(k|t): conditional probability of dependent variable = k given node t

subscript p: parent node

subscript r: child right node

subscript l: child left node

*P*_*l*_: probability in the left child node

*P*_*r*_: probability in the right child node (note: *P*_*l*_ + P_*r*_ = 1)

${x}_j^R$: best splitting value of variable *x*_*j*_

M: number of potential independent variables

K: level of dependent variables. For binary outcomes, K = 2

The larger the value of the improvement in impurity function, the greater difference between the two child nodes with respect to the prevalence of the dependent measure. The CART procedure selects the independent variable and the splitting cutoff of the continuous independent variable to maximize the improvement at each step. The tree grows as child nodes are divided into more child nodes. The terminal nodes are where predictions and inferences are made.

It is clear that different samples would produce different trees. One common way to assess how different the trees could be is using training and validation samples. To facilitate comparisons, the same set of training and validation samples were used in logistic regression model and CART model. In CART model, misclassification rates from both the training sample and the validation sample were compared to ensure the model is stable.

The maximum tree with the minimum misclassification error was examined and the misclassification error graph showed that it contained insignificant nodes, which reduced the misclassification error marginally but increased the complexity greatly. A popular stopping strategy was applied by predefining the minimum number of points in the terminal node to control the size of the tree [26]. The minimum node size was set to be 10% of the training sample size or about 150 subjects in our study. Models were assessed to identify a parsimonious tree that produces non-trivial results with acceptable misclassification rates.

## Results

### Logistic regression model

**Figure 1:**
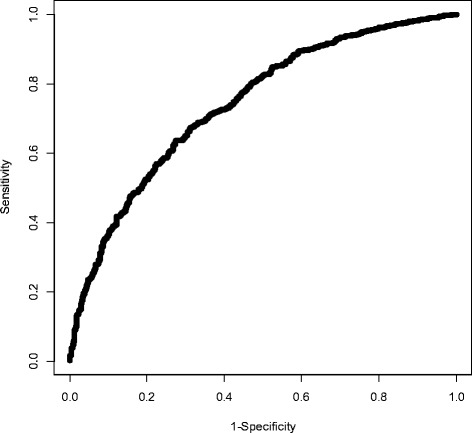
**ROC curve from logistic regression on the training sample.** Area under the curve = 0.7403.

The final model consisted of nine variables (Table 3). Males had the strongest association with being a cigarettes + ATP user vs. cigarettes-only user (adjusted OR 2.66, 95% CI 2.12 – 3.33). African Americans and Latino were more likely to be cigarettes + ATP users compared to whites (adjusted OR 1.58, 95% CI 1.21 – 2.07 and adjusted OR 1.52, 95% CI 1.16 – 1.99, respectively). Individuals with higher nicotine dependence were more likely to be cigarettes + ATP users (adjusted OR 1.51, 95% CI 1.20 – 1.90). Participants who buy their cigarettes were less likely to be cigarettes + ATP users compared to those who borrow cigarettes from others (adjusted OR 0.617, 95% CI 0.49 – 0.78). Individuals who were more sensitive to the price of cigarettes were more likely to be cigarettes + ATP users (adjusted OR 1.43, 95% CI 1.14 -1.79). Individuals who set limit on cigarettes per day were more likely to be cigarettes + ATP users (adjusted OR 1.30, 95% CI 1.04 – 1.62). Individuals with higher alcohol scores were more likely to be cigarettes + ATP users (adjusted OR 1.10, 95% CI 1.064-1.145). Older people were less likely to use cigarettes + ATPs (adjusted OR 0.97, 95% CI 0.96-0.98). Higher discrimination scores were associated with higher probability of using cigarettes + ATPs (adjusted OR 1.03, 95% CI 1.01 – 1.05). The C-index of the final model was 0.74, indicating good discriminatory capacity (Figure 1).

**Table 3:** **Results from logistic regression on the training cohort: parameter estimates and odds ratios**

**Parameter**	**Estimate**	**Odds ratio**	**95% CL for OR**	**P-value**
Intercept	−0.2617	NA	NA	0.3497
Age	−0.0265	0.974	(0.964, 0.983)	<.0001
Male	0.9766	2.655	(2.118, 3.329)	<.0001
Buy vs. Borrow	−0.4832	0.617	(0.486, 0.783)	<.0001
Alcohol	0.0986	1.104	(1.064, 1.145)	<.0001
Price influenced the brand they buy	0.3579	1.430	(1.144, 1.788)	0.0017
African American vs. white	0.4576	1.580	(1.208, 2.066)	0.0008
Latino vs. white	0.4170	1.517	(1.155, 1.994)	0.0028
Discrimination	0.0259	1.026	(1.007, 1.045)	0.0065
Time to first cig less than 30 min	0.4100	1.507	(1.197, 1.897)	0.0005
Limit cigarettes per day	0.2612	1.299	(1.041, 1.619)	0.0203

### Model validation

**Figure 2:**
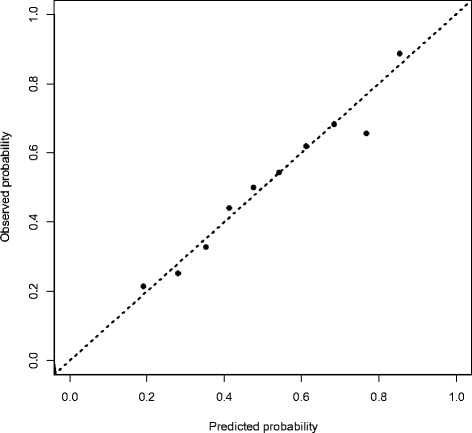
**Calibration plot from the validation sample.** Observed vs. predicted probabilities across deciles, *R* ^2^ = 0.96.

Participants were similar in terms of all profile characteristics (Table 2), except that participants in the validation cohort smoked about 1 cigarette per day less than the training cohort (10 vs. 9, p = 0.009). The model performed well in the validation cohort with good discrimination (c-index = 0.73) and excellent calibration with an intercept of 0.018 (p-value for difference from 0 = 0.65) and a slope = 0.96 (p-value for difference from 1 = 0.58). The R-square for the calibration regression was 0.96 and the Pearson correlation coefficient was 0.98 (p-value < 0.0001) (Figure 2).

### Classification and Regression Tree (CART) model

**Figure 3:**
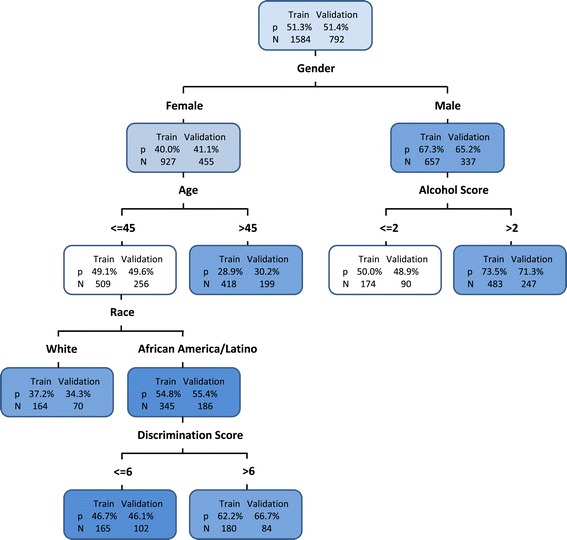
**Classification and Regression Tree model for predicting Cig + ATP users.** The number of participants (N) and the probability of Cig + ATP users (P) are given inside of each node for both training and validation samples.

Figure 3 shows the final tree results using the stopping rule of minimum node size no less than 150. The same independent training and validation samples were used as in the logistic regression. The misclassification rate was 0.342 for the training sample and 0.346 for the validation sample. The C-index was 0.70 for the training sample and 0.69 for the validation sample.

Males were more likely to be cigarettes + ATP users, especially when they were moderate to heavy drinkers (alcohol use score > 2). A male with a 3 or higher alcohol score had 73.5% probability of being a cigarettes + ATP user. Females were less likely to be cigarettes + ATP users, especially when they were older. Female participants aged 46 or older had a 29.0% probability of being cigarettes + ATP users. Among females age 45 years or younger, Latino and African Americans were more likely to be cigarettes + ATP users compared to whites. 37.2% of White females aged 45 years or younger were cigarettes + ATP users. Latino and African American females aged 45 or younger, who also experienced greater discrimination were more likely to be cigarettes + ATP users, about 62.2% probability if their discrimination score was greater than 6 (Figure 3). Interestingly, age, race, and discrimination effects that impacted female participants did not play important roles for males. Alcohol scores increased the risk of cigarettes + ATP use for males but were not important for females. These indicated informative interaction patterns to examine the profile characteristics of cigarettes + ATP users.

### Limitations

A hold-out validation strategy was used in this study to obtain independent training and validation datasets. The reduced data can result in an enlarged variance. Although this method is reasonable in this study because the sample size is large, other validation strategies, such as k-fold cross validation, which uses overlapped training data, may achieve more accurate performance estimation. We used a method suggested by Harrell [18] to trim potential models and then compared AIC of these potential models to obtain the final model. Other model selection strategies, such as LASSO and ridge regression were not compared with this method.

## Discussion

The CART model identified the five most important factors: gender, alcohol scores, age, race, and discrimination scores. The logistic regression model identified nine variables: the same five as the CART model, and additionally, whether the participant buys or borrows cigarettes from others, whether the participant limits cigarettes per day, price influences, and nicotine dependence. Therefore, the logistic regression model expanded the variable pool from the CART model.

The logistic regression model results in higher C-index than the CART model (0.74 versus 0.70 for the training sample and 0.73 versus 0.69 for the validation sample). However, the C-index from the CART model was not directly comparable to that in the logistic regression model because the classifiers varied across different subgroups in the CART model due to partial interaction effects. On the other hand, logistic regression models lack the ability to identify unspecified, complex inter-relationships between factors. In studies where interaction effects are unclear, it is impractical to test all potential interaction effects in logistic regression models. However, there might be potential inter-relationships, especially among demographic, psychosocial, and economic factors. Even if the logistic regression model achieves good model fit, we could still miss interaction effects that are significant to clinical practice. CART analysis is efficient to address these problems and it is easy to perform with available statistical software. It has great flexibility of building a model that can be easily interpreted through pictorial illustration, without pulling in too much complexity. CART can be considered as complementary to logistic regression models and the result from CART revealed clearly classified high-risk populations of ATP use among cigarette smokers.

## Conclusions

The growing trend of ATP use could ultimately cut down the effect of tobacco control efforts that we have seen in recent years. Compared to the traditional logistic regression model, our CART model is more straightforward in classifying individuals at high risk of using cigarettes + ATPs. This model identified fewer factors associated with cigarettes + ATP use and revealed partial interactions that are not easy to find in logistic regression, thus provided clearer direction for identification and treatment in clinical practice. In general, the CART methodology can be used to classify high risk or at need groups for identification for treatment protocols including behavioral interventions.

## Abbreviations

CART: Classification and Regression Trees
ATP: Alternative tobacco product
AIC: Akaike Information Criterion
